# The Analysis of Plantar Pressure Data Based on Multimodel Method in Patients with Anterior Cruciate Ligament Deficiency during Walking

**DOI:** 10.1155/2016/7891407

**Published:** 2016-12-06

**Authors:** Xiaoli Li, Hongshi Huang, Jie Wang, Yuanyuan Yu, Yingfang Ao

**Affiliations:** ^1^College of Electronic Information & Control Engineering, Beijing University of Technology, Beijing 100124, China; ^2^Institute of Sports Medicine, Peking University Third Hospital, Beijing 100191, China; ^3^School of Automation & Electronic Engineering, University of Science and Technology Beijing, Beijing 100083, China

## Abstract

The movement information of the human body can be recorded in the plantar pressure data, and the analysis of plantar pressure data can be used to judge whether the human body motion function is normal or not. A two-meter footscan® system was used to collect the plantar pressure data, and the kinetic and dynamic gait characteristics were extracted. According to the different description of gait characteristics, a set of models was established according to various people to present the movement of lower limbs. By the introduction of algorithm in machine learning, the FCM clustering algorithm is used to cluster the sample set and create a set of models, and then the SVM algorithm was used to identify the new samples, so as to complete the normal and abnormal motion function identification. The multimodel presented in this paper was carried out into the analysis of the anterior cruciate ligament deficiency. This method demonstrated being effective and can provide auxiliary analysis for clinical diagnosis.

## 1. Introduction

The foot is a very important part of the human body. Its basic function is to support the body, absorb impact force, produce forward thrust, and help regulate and maintain the balance of the human body. Under the action of gravity, the foot could get reaction force in the vertical direction by the ground when we stand or walk. When the structure or the state of the human body changes, plantar pressure distribution would change accordingly. The research of the human body based on static or dynamic plantar pressure data can reveal different characteristics of plantar pressure distribution comparing patients with normal controls, which help find the causes and related evolution.

The research of plantar pressure measurement was started by Beely in 1882. The systematic analysis of gait and extensive clinical research began in the 1950s. Eisenhardt et al. [1] studied the effects of different heel height of shoes on the foot bones of women after the study of plantar pressure of 30 women aged 18 to 30 who wore high heeled shoes. Eisenhardt et al. also pointed out that the normal human plantar pressure distribution had a certain pattern and foot deformity and dysfunction would damage the normal distribution of plantar pressure. Through the analysis of plantar pressure measurement, Minns and Craxford [2] got the difference of plantar pressure distribution between the patients with rheumatoid arthritis and the normal control. Abboud et al. [3] analyzed and compared the plantar pressure of diabetic patients and healthy person. Stokes et al. [4] pointed out that the pressure peak value caused by medial foot four toes decreased obviously when patient suffered from hallux valgus, and hallux valgus angle was related to plantar pressure deviation. Stolwijk et al. [5] explored that long time walking would change the walking pattern because of leg fatigue and led to the increasing burden of heel. Stolwijk et al. [6] compared and analyzed plantar pressure data from Malawi and the Netherlands with the analysis of the gait feature parameters of arch index (AI) and the trajectory of the center of pressure (COP) in order to explore why there was less foot disease in Malawi. Pataky and Maiwald [7] thought high quality biomechanical information was included in the plantar pressure data. For this reason, he developed 3D interactive visualization tools to analyze the plantar pressure data and explore foot behavior more deeply. Along with the development of sensor technology and the popularization of computer technology, the measurement and research of plantar pressure become more and more important in the field of gait research.

However, the plantar pressure data analysis is still far from mature. The current research work was mainly focused on the effect of human behavior on plantar pressure data and the relationship between plantar pressure and gait features [8, 9], and so on. On the other hand, due to the complex structure of the human body, the plantar pressure in different conditions also has complex performance. Large plantar pressure data will bring a heavy burden for medical workers. The intervention of relative algorithm in machine learning will make a great change to these situations.

Different classification criteria can generate different clustering analysis methods. With the development of the fuzzy set theory, Ruspini [10] introduced the concept of fuzzy partition in the cluster analysis at the end of the 1960s. And then many research results came out, such as clustering method based on similarity relation and fuzzy relation [11] and fuzzy clustering method based on the evolutionary algorithm [12]. The most widely used method is the fuzzy clustering method based on the objective function. This method can transform the clustering problem into a mathematical optimization problem, and it has been widely used due to its simple structure [13]. Among the fuzzy clustering algorithms based on objective function, the fuzzy *C* mean clustering algorithm (FCM) created by Bezdek [14] and developed by Dunn is the most typical representative one. This algorithm is derived by the optimization of the objective function of hard clustering, and it is produced by using the concept of fuzzy partition.

On the other hand, support vector machine (SVM) algorithm which is based on statistical learning is playing a key role in pattern recognition. Support vector machine is a machine learning algorithm developed in the 1990s, which was first proposed by Cortes and Vapnik [15] in 1995. It is a supervised learning algorithm. Based on statistical learning theory, by seeking structural minimum risk to enhance its learning generalization ability, SVM can obtain better statistical learning results even in the case of limited sample information.

Multimodel adaptive control [16, 17] is a major control method of control field in the past 20 years. By dividing the model parameters into multiple subsections, multiple models of the system according to different subsection will be set up. The whole model of the system can be got by the different models in different subsections by using different ways of combinations.

With the advent of new plantar pressure measurement devices, a large amount of plantar pressure data is measured, but these data have not been adequately utilized. Currently, medical diagnostic work is done entirely by doctors relying on their own experience. In this paper, based on FCM clustering algorithm and SVM algorithm, the concept of multimodel is applied to the classification and identification of anterior cruciate ligament deficiency. This study introduces machine learning algorithm into clinical diagnosis creatively which can bring great help for the diagnosis and rehabilitation of clinical medicine.

## 2. Multimodel Modeling of Plantar Pressure Characteristics

In this study, we used a plantar pressure measurement device called footscan to measure plantar pressure data. Anterior cruciate ligament deficiency was regarded as the main analysis object. All subjects included both normal subjects and anterior cruciate ligament rupture subjects. We will first put all the data samples together for analysis, with FCM algorithm for clustering, and SVM algorithm for identification. Then, with the aid of prior knowledge, the left and right plantar pressure data were analyzed, respectively, in the same way.

### 2.1. Plantar Pressure Data Feature Modeling

**Figure 1:**
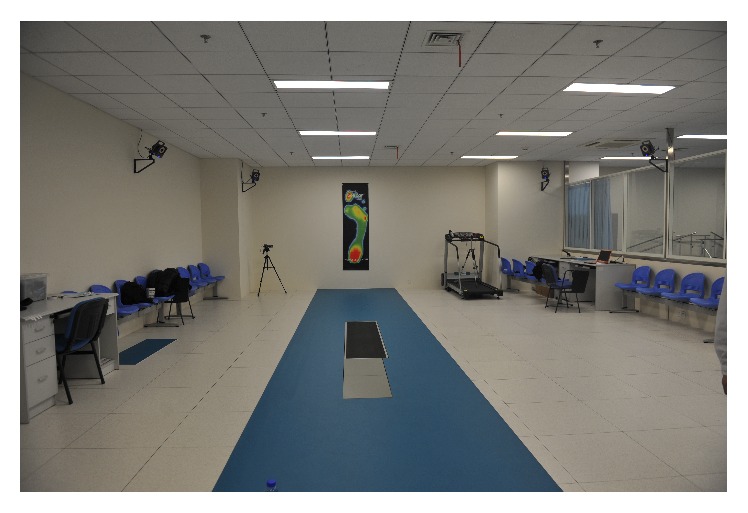
Footscan device.

As shown in Figure 1, footscan plantar pressure measurement system will be used to collect the plantar pressure data of the test subjects. This system can be used to measure plantar pressure in static or dynamic motion. The total size of measurement is 200 cm *∗* 40 cm, and there are 16384 pressure sensors. It is placed in the center of a 16-meter long runway. During the test, testers walk barefoot through the measurement according to their own walking habits. Testers must follow the “three-step” protocol; in other words, testers need to walk alternating their left and right foot. In order to ensure the validity of the measurement data, each person's foot landing process should begin with the first touchdown of heel. The sampling frequency is set to 126 Hz.

Gait characteristics used in biomechanics and sports medicine always play important roles in the analysis of plantar pressure data, such as foot progression angle, AI, and the trajectory of COP. Among these, the trajectory of COP will play a crucial role in the analysis of plantar pressure, especially for the anterior cruciate ligament deficiency.

**Figure 2:**
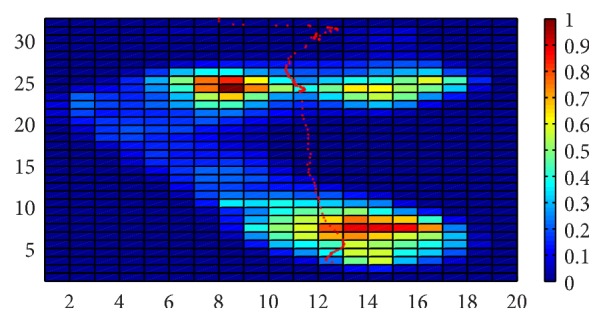
The trajectory of the center of pressure.

The trajectory of COP [5] is a parameter that is used to describe the movement of center of foot gravity in the whole stance phase. As given in paper [18], this parameter can be obtained by connecting pressure centers of all pressure data frames into a line. An example of COP can be seen in Figure 2.

### 2.2. Fuzzy *C*-Means Clustering Algorithm

Fuzzy *C*-means clustering (FCM) algorithm is a fuzzy clustering algorithm. Data samples are allowed to belong to two or more clusters in this algorithm, and membership degree was used to determine which cluster it belongs to. By minimizing the objective function based on a certain norm and clustering prototype, the FCM algorithm attempts to divide a finite collection of elements into a collection of fuzzy clusters.

*X* is set as a series of samples:

(1) $$\begin{array}{c} X=\left\{\;x_{1},x_{2},\ldots ,x_{n}\;\right\}\subset R^{s}, \end{array}$$

where *n* is the number of samples and *s* is the dimension of the sample space. When *c* (*c* > 1) is set as the number of clusters, FCM can be described as below

(2) min JFCMU,V=∑i=1c∑j=1nuijmxj−vi2 ∑i=1cuij=1,1≤j≤n ∑j=1nuij>0,1≤i≤c uij≥0,1≤i≤c,  1≤j≤n,

where

(3) $$\begin{array}{c} u_{ij}={\left[\;{\sum_{r=1}^{c}\left(\frac{\left\|x_{j}-v_{i}\right\|}{\left\|x_{j}-v_{r}\right\|}\right)}^{2/\left(m-2\right)}\;\right]}^{-1} \end{array}$$

In this formula, *m* is the weighted index; *U* is fuzzy membership matrix:

(4) $$\begin{array}{c} U={\left[\;u_{ij}\;\right]}_{1\leq i\leq c,1\leq j\leq n}. \end{array}$$

*V* is a matrix of clustering centers:

(5) $$\begin{array}{c} V=\left[\;v_{1},v_{2},\ldots ,v_{c}\;\right]. \end{array}$$

In this algorithm, clustering centers can be calculated by the following formula:

(6) $$\begin{array}{c} v_{i}=\frac{\sum_{j=1}^{n}u_{ij}^{m}x_{j}}{\sum_{j=1}^{n}u_{ij}^{m}},\;i=1,2,\ldots ,c. \end{array}$$

The FCM algorithm can be realized by using the following steps.

**Step 1 .**

Choose the number of clusters. Initialize the fuzzy membership matrix. The membership values are randomly generated.

**Step 2 .**

Calculate the clustering centers using the formula above.

**Step 3 .**

Update the membership matrix. If

(7) $$\begin{array}{c} \left\|\;U^{k+1}-U^{k}\;\right\|<\varepsilon \end{array}$$

then stop. Otherwise return to Step 2.

*K*-means clustering algorithm [19] is the most well-known by scholars. FCM is similar to the *K*-means algorithm. The algorithm minimizes intracluster variance as well but has the same problems as *K*-means. FCM is sensitive to the initial choice of the fuzzy membership matrix, and the result may not be the global minimum sometimes.

### 2.3. Multimodel Set Based on Plantar Pressure Data and FCM Algorithm

In this section, FCM algorithm is used to classify different plantar pressure data samples. According to the important parameter COP of gait characteristics, multiple models will be set up for different testers.

First of all, the plantar pressure data of left or right side is different. On the other hand, the plantar pressure data on each side will include normal foot data and abnormal foot data. Therefore, the plantar pressure data models will have four types. As mentioned above, the plantar pressure data can be collected by footscan device. And each data sample is presented by a set of data tables sorted in chronological order. Each frame of pressure data can be described by a data table whose rows and columns are equal to sensors array on footscan plate. The number of tables depends on the moving speed of tester and the sampling frequency.

Firstly, the pressure center of the two-dimensional pressure data table can be calculated according to the following formula:

(8) Xc=∑Pixi∑PiYc=∑Piyi∑Pi.

In this formula, *X* and *Y* represent the coordinate values of the two dimensions. And the lower left corner of the space occupied by the foot is recorded as the origin of the coordinate. *P*_*i*_ indicates the pressure value of each point. The pressure centers of each frame were connected together, and then COP line can be got.

Because different person have different foot size, if the COP lines are compared directly, the result must be inaccurate. Therefore, these plantar pressure data also need a coordinate mapping in the following formula:

(9) Xc′=30XcLXYc′=20YcLY.

In this formula, *X*_*c*′_ and *Y*_*c*′_ are the pressure center coordinate value after coordinate mapping; *L*_*X*_ and *L*_*Y*_ are the width of the two dimensions. Through this formula, all the plantar pressure data will be mapped to a 30*∗*20 (this value is derived from the sensor array model corresponding to the standard foot size) rectangular coordinate system.

Because each COP line is composed of hundreds of pressure center points, if all the data is used for cluster analysis, it will lead to a big computing burden. In order to reduce the computational complexity, the extraction of the coordinate points should be conducted. In our study, 5 key points (10 characteristic values) will be extracted for characteristic description.

In order to guarantee the same influence of *X* and *Y* coordinate in analysis process, the extracted features need to be normalized. The feature values will be transformed into the [0,1] range by dividing the maximum value of the corresponding feature in all the samples.

**Figure 3:**
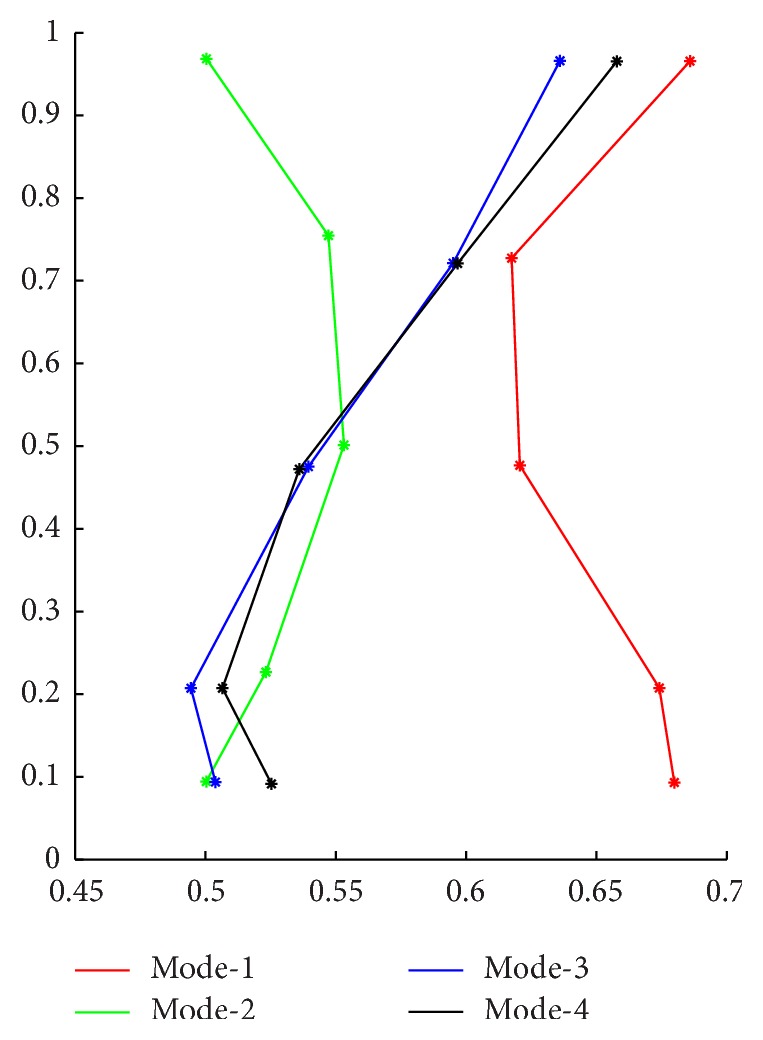
Plantar pressure data model of double sides.

**Figure 4 fig4:**
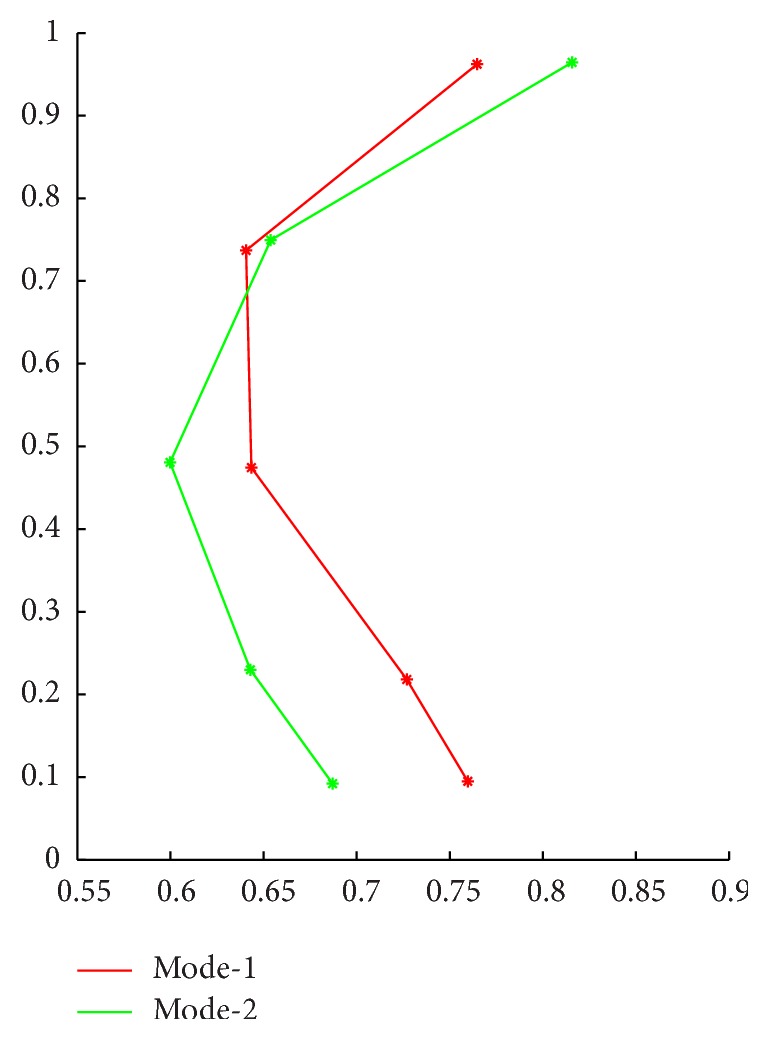
Plantar pressure data model of left side.

**Figure 5:**
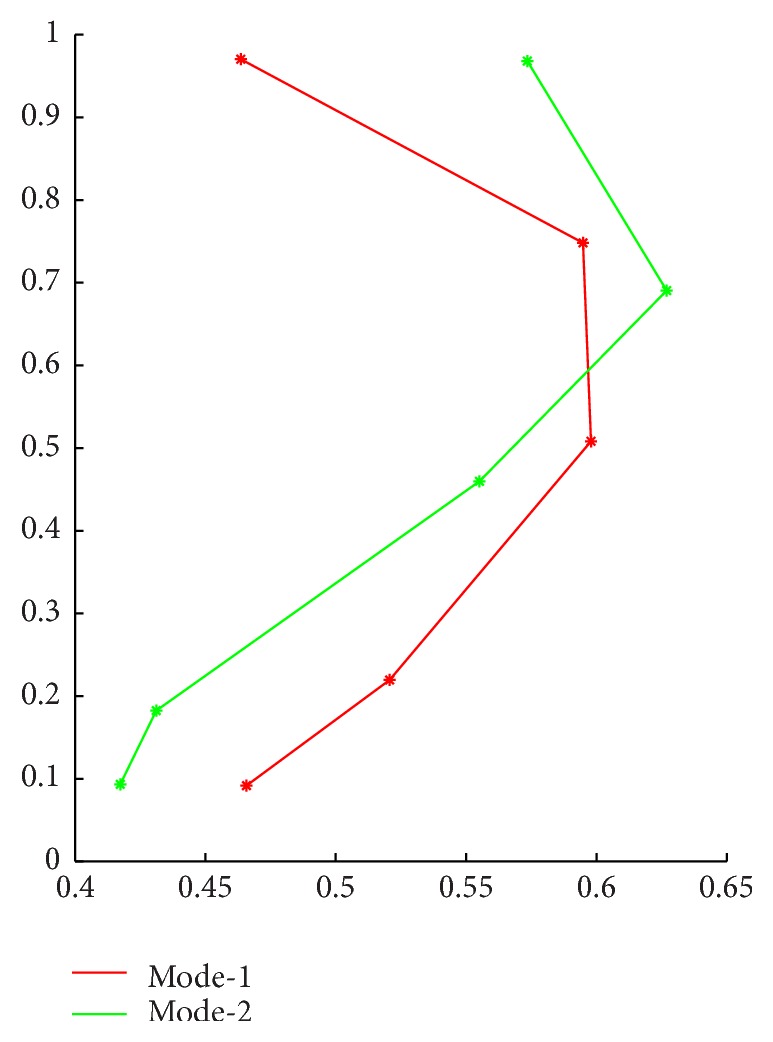
Plantar pressure data model of right side.

By using FCM and normalization process above, the plantar pressure data can be divided into different models. The model set with four and two models will be shown as in Figures 3 [Fig fig4]–5.

## 3. Diagnose of Sports Injuries Based on Multimodel

According to the results of the above clustering analysis, different plantar pressure data can be classified as different COP model. Once the model set was set up, how to classify a new data sample to model set will be the main problem. The following SVM algorithm will be used to solve this problem.

### 3.1. Support Vector Machine

SVM is an important algorithm in machine learning; it may always be used for classification and regression analysis. It was first proposed for linear two-class classification problem by constructing a hyperplane to separate two kinds of samples. Now the method of SVM has been extended to nonlinear classification problem by using the penalty function and kernel function.

#### 3.1.1. Linear Classification Problem

For a set of training samples (*x*_*i*_, *y*_*i*_), *i* = 1,2,…, *n*, *x*_*i*_ ∈ *R* ^*N*^, *y*_*i*_ ∈ {−1,1}; *x*_*i*_ represents the sample vector of dimension *N* and *y*_*i*_ represents the category. Linear classifier is designed to achieve such a classification of *N* dimensional space and the hyperplane equation can be expressed as *ω* ^*T*^ *x* + *b* = 0, *ω* ∈ *R* ^*N*^, *b* ∈ *R*.

**Figure 6:**
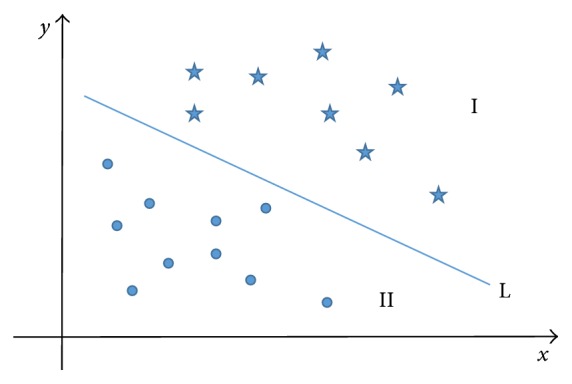
Linear classification of two-dimensional space.

Taking the two-dimensional plane as an example, two different groups of points on the plane are separated by a straight line L (the hyperplane in two-dimensional space is a straight line), which is shown in Figure 6.

(10) $$\begin{array}{c} f\left(\;x\;\right)=\mathrm{sgn}\left(\;\omega^{T}x+b\;\right). \end{array}$$

If *f*(*x*) = 0, then *x* is a point in the hyperplane; if *f*(*x*) = 1, *x* is a point in mode I; otherwise, if *f*(*x*) = −1, *x* is a point in mode II.

In this way, we only need to get the value of *ω* and *b* to construct the classification function.

Finally the design of the classifier can be transformed into a convex optimization problem as follows:

(11) min 12ω2s.t. yiωTxi+b≥1,i=1,2,…,n.

The convex optimization problem can be converted to the following function expression by introducing the Lagrange duality factor *α*:

(12) $$\begin{array}{c} L\left(\;\omega ,b,\alpha \;\right)=\frac{1}{2}{\left\|\;\omega \;\right\|}^{2}-\overset{n}{\underset{i=1}{\sum }}\alpha_{i}\left(\;y_{i}\left(\;\omega^{T}x_{i}+b\;\right)-1\;\right). \end{array}$$

And then the objective function becomes

(13) minω,b θω=minω,b ⁡maxαi≥0 ⁡Lω,b,α.

#### 3.1.2. Nonlinear Classification Problem

As to nonlinear classification problem, the support vector machine maps the sample data to the high dimension space by kernel function, so that the nonlinear classification problem can be solved.

Now the classification function can be expressed as

(14) $$\begin{array}{c} f\left(\;x\;\right)=\overset{n}{\underset{i=1}{\sum }}\alpha_{i}y_{i}\kappa \left(\;x_{i},x\;\right)+b. \end{array}$$

*α*_*i*_ can be obtained by solving the following problems:

(15) maxα ∑i=1nαi−12αiαjyiyjκxi,xjs.t. αi≥0,i=1,2,…,n ∑i=1nαiyi=0.

And then according to the following formula, *ω* and *b* can be calculated:

(16) ω=∑i=1nαiyixib=−ω·∑i=1nαixi2∑yi=1αi.

SVM is a supervised learning algorithm. The application of this algorithm is divided into two parts: a set of data for classifier training and the new sample data for identification. The classifier training is progress of calculating *α*, *ω*, and *b* in order to determine the classification function and the identification process is to bring the new samples into the classification function to calculate the category.

There are some commonly used kernel functions:

 Polynomial kernel function: *κ*(*x*_1_, *x*_2_) = (〈*x*_1_, *x*_2_〉 + *R*)^*d*^.
 Gauss kernel function: *κ*(*x*_1_, *x*_2_) = exp⁡{−‖*x*_1_ − *x*_2_‖^2^/2*σ* ^2^}.
 Linear kernel function: *κ*(*x*_1_, *x*_2_) = 〈*x*_1_, *x*_2_〉.

### 3.2. Multimodel Identification for Plantar Pressure Based on SVM

SVM algorithm is a supervised learning algorithm. After the plantar pressure data has been classified by FCM algorithm, all the plantar pressure data and the models to which these data belong can be got. The pressure data and the models to which these data belong will be used to train parameters *α*, *ω*, and *b*. Once these parameters have been trained convergent, function (14) will be used to decide which model it belongs to when new plantar data is given.

## 4. Simulation

Anterior cruciate ligament is a very important structure of knee joint of human body. It can be used to maintain the stability of knee motion. Anterior cruciate ligament deficiency (ACLD) is a kind of common sports injury. When a man got ACLD, the injury will lead to instability of the knee and has a serious negative impact on the knee function. And it will lead to the abnormality of COP line [20–24].

Through the data processing, we get 100 groups of plantar pressure data characteristic sequence, including the normal data and ACLD data. We will use FCM algorithm to do clustering analysis firstly and then use the SVM algorithm to do the data identification.

Firstly, we put left and right side data together for analysis. These 100 sets of data should include 4 models: the left side of the normal data, the left side of the ACLD data, the right side of the normal data, and the right side of the ACLD data. So the number of clusters in FCM algorithm is set to *c* = 4. Then the weighted index is set to *m* = 2; maximum iteration number is set to 100; the minimum iteration error is set to *ε* = 0.00001. The cluster analysis results are shown in Table 1.

In Table 1, *C*_1*x*_ represents the *X*-coordinate value of the first feature point in COP line; the others are similar. Mode_1 indicates the sample's membership to Mode_1 which is shown in Figure 3. Category represents the final category to which current sample belongs.

The results of the FCM clustering analysis are used for the training and identification of the SVM algorithm. We select 80 groups of data in the front as training samples directly, 20 sets of data left as the test samples. The accuracy of final identification results is 50%. The accuracy was evaluated over the cluster labels. There is still a gap of the identification accuracy with our expectations.

Next, we analyze the plantar pressure data of the left and right sides, respectively. Take the left side data as an example. At this time all data samples should contain two categories, the normal data and the ACLD data, so the number of clusters in FCM is set to *c* = 2, the other parameters unchanged. The results of cluster analysis are shown in Table 2.

In Table 2, Mode_1 and Mode_2 are shown in Figure 4. We use the *K*-cross validation method (we take *K* = 5 in this study) for the analysis of the data. These data were divided into five groups. Because the original data sequence is randomly arranged, every 10 adjacent samples were divided into the same group. Four groups are selected as the training data and the remaining set of data as the test data for analysis. The final identification results are calculated by the SVM algorithm, which is shown in Table 3.

The average value of the five simulation test results is obtained, and the average identification accuracy is 76%.

Then we can use the same method to analyze the right plantar pressure data and get the result of FCM cluster analysis which is shown in Table 4.

In Table 4, Mode_1 and Mode_2 are shown in Figure 5. All the data were divided into five groups; four groups were selected as the training data and the remaining set of data as the test data. The final identification results are shown in Table 5.

The average value of the five simulation test results is obtained, and the average identification accuracy is 62%.

**Figure 7:**
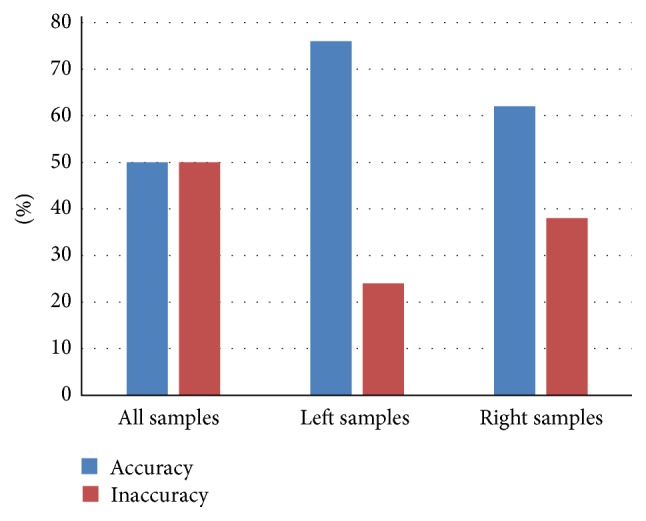
Comparison of identification results of several sets of analyses.

Three groups of experiments were done in this paper. In the first experiment, 100 sets of data samples were divided into 4 categories, with FCM algorithm for clustering and SVM algorithm for identification. The final identification accuracy is 50%. In the second and third experiments, the left and right plantar pressure data were analyzed, respectively, in the same way. The final identification accuracy is 76% and 62%. The comparison of identification results is shown in Figure 7.

From the simulation, it can be seen that the cluster and identification results on plantar pressure data analysis are acceptable by using the FCM and SVM machine learning algorithm. On the other hand, when the number of models in model sets is larger, identification accuracy will be decreased. If we use existing information or knowledge to reduce the number of models in model set, the accuracy can be improved obviously. Compared with the doctor's clinical diagnosis, the results of accuracy rate can be accepted, but the accuracy of this method should be improved in the future study. This research can be used as a reference for clinical diagnosis of doctors and provide an auxiliary analysis.

## 5. Conclusion

The machine learning algorithm is applied to the analysis of plantar pressure data. The COP line is used as the main feature to describe characteristics to complete the identification of the ACLD patient and the normal control. FCM algorithm is used for the samples cluster, and the SVM algorithm is used for the identification. To the authors' knowledge, there is still no research examining the plantar pressure using the clustering algorithm. The combination of machine learning algorithm with plantar pressure data analysis is a tentative research; the result in this paper is acceptable, but the accuracy should be improved in future study. This research can be an auxiliary method for doctors and has great significance for clinical diagnosis, rehabilitation evaluation, orthosis prescription, and sports exercises.

## Figures and Tables

**Table 1:** Results of cluster analysis of plantar pressure data model from double sides.

Number	*C*_1*x*_	*C*_1*y*_	*C*_2*x*_	*C*_2*y*_	*C*_3*x*_	*C*_3*y*_	*C*_4*x*_	*C*_4*y*_	*C*_5*x*_	*C*_5*y*_	Mode_1	Mode_2	Mode_3	Mode_4	Category
1	0.07	0.75	0.18	0.75	0.86	0.88	0.93	0.94	0.93	0.88	0.29	0.19	0.31	0.22	3
2	0.11	0.69	0.14	0.75	0.61	0.75	0.82	0.75	0.96	0.88	0.30	0.15	0.37	0.18	3
3	0.07	0.81	0.11	0.81	0.36	0.81	0.82	0.75	0.96	0.88	0.13	0.17	0.12	0.59	4
4	0.11	0.81	0.18	0.81	0.54	0.69	0.82	0.75	1.00	0.81	0.16	0.33	0.19	0.33	4
5	0.11	0.81	0.18	0.81	0.50	0.69	0.79	0.69	1.00	0.69	0.18	0.17	0.14	0.51	4
6	0.11	0.75	0.18	0.81	0.46	0.69	0.79	0.69	0.96	0.69	0.22	0.25	0.20	0.33	4
7	0.11	0.81	0.14	0.81	0.46	0.63	0.79	0.63	1.00	0.81	0.15	0.38	0.19	0.28	2
8	0.11	0.81	0.21	0.81	0.54	0.50	0.75	0.44	0.96	0.44	0.21	0.28	0.19	0.32	4
9	0.11	0.81	0.18	0.81	0.50	0.69	0.79	0.69	1.00	0.69	0.18	0.17	0.14	0.51	4
10	0.11	0.75	0.18	0.81	0.46	0.69	0.79	0.69	0.96	0.69	0.22	0.25	0.20	0.33	4
11	0.11	0.75	0.18	0.81	0.36	0.69	0.75	0.56	1.00	0.75	0.14	0.24	0.16	0.46	4
12	0.11	0.63	0.18	0.56	0.43	0.56	0.75	0.63	0.96	0.94	0.42	0.12	0.31	0.14	1
13	0.07	0.81	0.32	0.63	0.46	0.63	0.39	0.63	0.93	0.81	0.22	0.24	0.18	0.36	4
14	0.07	0.56	0.14	0.56	0.29	0.56	0.75	0.56	0.96	0.94	0.31	0.15	0.34	0.20	3
15	0.11	0.56	0.18	0.63	0.21	0.63	0.82	0.81	1.00	0.88	0.31	0.13	0.38	0.18	3
16	0.11	0.50	0.14	0.56	0.29	0.56	0.79	0.75	0.96	0.94	0.40	0.10	0.34	0.16	1
17	0.11	0.50	0.18	0.56	0.46	0.50	0.79	0.63	1.00	1.00	0.36	0.15	0.31	0.17	1
18	0.11	0.75	0.29	0.69	0.57	0.56	0.75	0.63	0.96	0.81	0.22	0.26	0.19	0.33	4
19	0.07	0.81	0.25	0.75	0.46	0.63	0.79	0.63	0.96	0.81	0.41	0.11	0.26	0.22	1
20	0.11	0.81	0.29	0.69	0.68	0.50	0.75	0.56	0.96	0.81	0.19	0.15	0.14	0.52	4
21	0.11	0.75	0.36	0.63	0.61	0.56	0.75	0.56	0.96	0.75	0.47	0.10	0.27	0.16	1
22	0.11	0.88	0.18	0.88	0.46	0.75	0.21	0.88	0.96	0.56	0.20	0.27	0.18	0.35	4
23	0.07	0.94	0.18	0.94	0.25	0.94	0.82	0.63	0.96	0.50	0.14	0.29	0.16	0.41	4
24	0.11	0.94	0.29	0.81	0.50	0.56	0.36	0.69	1.00	0.44	0.21	0.22	0.17	0.39	4
25	0.11	0.88	0.18	0.94	0.57	0.63	0.79	0.56	0.93	0.63	0.11	0.17	0.11	0.61	4
26	0.07	0.75	0.14	0.75	0.39	0.75	0.71	0.69	0.96	0.88	0.16	0.21	0.16	0.47	4
27	0.07	0.69	0.32	0.56	0.54	0.56	0.75	0.56	0.96	0.94	0.18	0.15	0.14	0.53	4
28	0.07	0.63	0.21	0.56	0.39	0.56	0.75	0.63	1.00	0.94	0.27	0.17	0.43	0.13	3
29	0.11	0.63	0.39	0.44	0.54	0.56	0.75	0.56	1.00	0.94	0.16	0.39	0.21	0.24	2
30	0.11	0.69	0.21	0.69	0.50	0.56	0.71	0.56	0.96	0.81	0.24	0.21	0.20	0.35	4
31	0.11	0.63	0.39	0.44	0.54	0.56	0.75	0.56	1.00	0.94	0.16	0.39	0.21	0.24	2
32	0.11	0.63	0.39	0.38	0.46	0.44	0.71	0.50	0.96	0.94	0.38	0.13	0.37	0.13	1
33	0.07	0.63	0.25	0.63	0.43	0.56	0.71	0.50	0.96	0.88	0.20	0.15	0.15	0.50	4
34	0.14	0.75	0.18	0.81	0.46	0.69	0.71	0.69	0.96	0.81	0.16	0.18	0.15	0.51	4
35	0.07	0.69	0.18	0.75	0.46	0.75	0.75	0.75	0.96	0.88	0.41	0.12	0.29	0.17	1
36	0.11	0.81	0.14	0.88	0.36	0.88	0.82	0.75	1.00	0.81	0.15	0.33	0.18	0.35	4
37	0.11	0.69	0.18	0.69	0.43	0.75	0.79	0.81	1.00	0.88	0.30	0.15	0.36	0.19	3
38	0.07	0.75	0.14	0.81	0.36	0.75	0.75	0.75	0.96	0.94	0.13	0.17	0.13	0.57	4
39	0.07	0.81	0.18	0.81	0.43	0.75	0.79	0.75	1.00	0.88	0.18	0.21	0.18	0.43	4
40	0.07	0.81	0.25	0.69	0.39	0.63	0.75	0.63	0.93	0.69	0.16	0.27	0.18	0.39	4
41	0.07	0.81	0.29	0.63	0.43	0.56	0.71	0.44	0.82	0.56	0.22	0.26	0.21	0.31	4
42	0.11	0.81	0.21	0.69	0.50	0.56	0.82	0.56	0.86	0.56	0.21	0.27	0.20	0.32	4
43	0.11	0.88	0.29	0.75	0.50	0.63	0.75	0.56	0.86	0.56	0.23	0.27	0.22	0.28	4
44	0.04	0.81	0.21	0.75	0.54	0.44	0.75	0.50	0.86	0.63	0.38	0.15	0.31	0.16	1
45	0.07	0.56	0.18	0.56	0.54	0.63	0.79	0.75	0.96	0.81	0.29	0.21	0.32	0.19	3
46	0.11	0.56	0.50	0.38	0.68	0.44	0.75	0.63	0.96	0.75	0.25	0.19	0.41	0.14	3
47	0.07	0.63	0.29	0.44	0.50	0.50	0.75	0.69	0.96	0.81	0.44	0.11	0.26	0.19	1
48	0.07	0.56	0.29	0.44	0.64	0.50	0.75	0.63	0.96	0.81	0.45	0.10	0.26	0.19	1
49	0.11	0.69	0.21	0.69	0.50	0.50	0.71	0.56	0.96	0.81	0.17	0.14	0.13	0.55	4
50	0.11	0.63	0.18	0.63	0.54	0.44	0.75	0.63	1.00	0.88	0.45	0.10	0.26	0.19	1
51	0.11	0.50	0.18	0.44	0.50	0.50	0.25	0.44	0.96	0.38	0.39	0.13	0.31	0.16	1
52	0.11	0.63	0.14	0.50	0.36	0.50	0.79	0.31	0.96	0.31	0.17	0.42	0.17	0.24	2
53	0.07	0.50	0.14	0.50	0.43	0.50	0.75	0.56	0.96	0.44	0.34	0.20	0.32	0.14	1
54	0.07	0.31	0.14	0.25	0.39	0.44	0.79	0.56	1.00	0.56	0.37	0.16	0.34	0.13	1
55	0.07	0.25	0.14	0.25	0.46	0.50	0.79	0.63	0.96	0.56	0.40	0.14	0.26	0.20	1
56	0.11	0.31	0.18	0.25	0.18	0.25	0.79	0.63	1.00	0.63	0.35	0.18	0.31	0.16	1
57	0.11	0.25	0.14	0.25	0.57	0.56	0.82	0.56	1.00	0.56	0.32	0.14	0.38	0.17	3
58	0.11	0.31	0.14	0.31	0.46	0.50	0.79	0.56	1.00	0.50	0.26	0.22	0.40	0.12	3
59	0.07	0.25	0.14	0.25	0.46	0.50	0.79	0.63	0.96	0.56	0.40	0.14	0.26	0.20	1
60	0.11	0.31	0.18	0.25	0.18	0.25	0.79	0.63	1.00	0.63	0.35	0.18	0.31	0.16	1
61	0.11	0.31	0.14	0.25	0.43	0.44	0.79	0.56	1.00	0.63	0.41	0.15	0.27	0.17	1
62	0.14	0.44	0.18	0.44	0.43	0.50	0.75	0.50	0.82	0.56	0.34	0.14	0.37	0.14	3
63	0.07	0.44	0.21	0.38	0.57	0.44	0.79	0.38	0.93	0.19	0.14	0.52	0.17	0.17	2
64	0.11	0.56	0.14	0.56	0.50	0.50	0.75	0.38	0.96	0.25	0.12	0.56	0.16	0.15	2
65	0.07	0.31	0.18	0.31	0.46	0.38	0.75	0.44	0.96	0.25	0.26	0.20	0.40	0.14	3
66	0.11	0.38	0.14	0.38	0.43	0.44	0.75	0.50	0.96	0.31	0.13	0.52	0.14	0.21	2
67	0.11	0.44	0.29	0.50	0.57	0.50	0.75	0.56	0.96	0.38	0.29	0.14	0.43	0.14	3
68	0.07	0.50	0.25	0.63	0.43	0.63	0.79	0.63	1.00	0.44	0.14	0.33	0.15	0.39	4
69	0.07	0.44	0.14	0.50	0.54	0.56	0.32	0.56	0.96	0.38	0.23	0.23	0.20	0.34	4
70	0.11	0.44	0.32	0.50	0.54	0.50	0.79	0.44	1.00	0.25	0.10	0.63	0.13	0.13	2
71	0.11	0.56	0.32	0.75	0.57	0.75	0.79	0.56	0.96	0.31	0.11	0.61	0.15	0.14	2
72	0.11	0.25	0.18	0.25	0.50	0.38	0.18	0.25	0.96	0.56	0.26	0.20	0.39	0.15	3
73	0.11	0.44	0.18	0.44	0.46	0.63	0.82	0.81	0.96	0.88	0.27	0.19	0.41	0.13	3
74	0.07	0.31	0.18	0.31	0.36	0.38	0.21	0.31	0.96	0.50	0.39	0.13	0.27	0.21	1
75	0.11	0.38	0.14	0.38	0.25	0.38	0.14	0.38	0.96	0.50	0.27	0.19	0.39	0.15	3
76	0.07	0.25	0.29	0.50	0.61	0.63	0.75	0.63	0.96	0.50	0.30	0.20	0.33	0.17	3
77	0.11	0.44	0.14	0.44	0.54	0.56	0.71	0.56	0.96	0.44	0.21	0.14	0.17	0.49	4
78	0.07	0.38	0.18	0.38	0.61	0.63	0.75	0.56	0.96	0.50	0.29	0.16	0.39	0.15	3
79	0.11	0.38	0.25	0.50	0.64	0.56	0.79	0.56	0.96	0.50	0.25	0.25	0.36	0.13	3
80	0.07	0.38	0.25	0.50	0.61	0.63	0.75	0.63	0.96	0.50	0.16	0.44	0.17	0.24	2
81	0.07	0.38	0.25	0.44	0.46	0.44	0.82	0.50	0.96	0.50	0.29	0.21	0.33	0.17	3
82	0.11	0.25	0.18	0.25	0.50	0.44	0.79	0.56	0.96	0.69	0.28	0.21	0.38	0.12	3
83	0.07	0.25	0.25	0.38	0.43	0.38	0.82	0.50	0.96	0.56	0.17	0.42	0.19	0.22	2
84	0.11	0.31	0.29	0.44	0.50	0.50	0.82	0.50	0.96	0.56	0.08	0.70	0.10	0.12	2
85	0.07	0.38	0.18	0.38	0.54	0.44	0.79	0.50	0.96	0.50	0.11	0.61	0.14	0.14	2
86	0.07	0.25	0.18	0.31	0.43	0.38	0.75	0.56	0.96	0.50	0.11	0.59	0.15	0.15	2
87	0.11	0.31	0.18	0.38	0.43	0.50	0.79	0.63	0.96	0.63	0.25	0.18	0.47	0.10	3
88	0.07	0.44	0.21	0.44	0.57	0.56	0.50	0.63	0.96	0.38	0.21	0.27	0.32	0.19	3
89	0.07	0.50	0.39	0.69	0.57	0.63	0.75	0.56	0.96	0.38	0.22	0.27	0.18	0.33	4
90	0.11	0.44	0.18	0.50	0.57	0.56	0.75	0.56	0.96	0.31	0.17	0.36	0.18	0.29	2
91	0.07	0.38	0.21	0.44	0.46	0.56	0.75	0.56	0.96	0.25	0.15	0.42	0.17	0.27	2
92	0.07	0.38	0.25	0.50	0.57	0.56	0.75	0.50	0.96	0.25	0.13	0.56	0.16	0.16	2
93	0.11	0.44	0.21	0.50	0.61	0.63	0.79	0.56	1.00	0.38	0.19	0.31	0.19	0.31	2
94	0.07	0.38	0.14	0.38	0.46	0.50	0.79	0.56	1.00	0.50	0.35	0.19	0.30	0.16	1
95	0.07	0.44	0.14	0.44	0.36	0.44	0.75	0.56	0.96	0.44	0.41	0.14	0.30	0.15	1
96	0.11	0.56	0.21	0.56	0.43	0.63	0.75	0.63	0.96	0.38	0.25	0.25	0.37	0.13	3
97	0.11	0.44	0.21	0.44	0.54	0.56	0.79	0.50	0.96	0.44	0.08	0.70	0.10	0.12	2
98	0.11	0.38	0.21	0.38	0.61	0.50	0.82	0.44	1.00	0.38	0.16	0.46	0.19	0.18	2
99	0.11	0.38	0.18	0.38	0.57	0.56	0.75	0.63	0.96	0.31	0.26	0.16	0.45	0.13	3
100	0.11	0.44	0.29	0.50	0.54	0.63	0.79	0.69	1.00	0.50	0.12	0.55	0.15	0.18	2

**Table 2:** Results of cluster analysis of plantar pressure data model from left side.

Number	*C*_1*x*_	*C*_1*y*_	*C*_2*x*_	*C*_2*y*_	*C*_3*x*_	*C*_3*y*_	*C*_4*x*_	*C*_4*y*_	*C*_5*x*_	*C*_5*y*_	Mode_1	Mode_2	Category
1	0.07	0.75	0.18	0.75	0.86	0.88	0.93	0.94	0.93	0.88	0.46	0.54	2
2	0.11	0.69	0.14	0.75	0.61	0.75	0.82	0.75	0.96	0.88	0.37	0.63	2
3	0.07	0.81	0.11	0.81	0.36	0.81	0.82	0.75	0.96	0.88	0.71	0.29	1
4	0.11	0.81	0.18	0.81	0.54	0.69	0.82	0.75	1.00	0.81	0.65	0.35	1
5	0.11	0.81	0.18	0.81	0.50	0.69	0.79	0.69	1.00	0.69	0.68	0.32	1
6	0.11	0.75	0.18	0.81	0.46	0.69	0.79	0.69	0.96	0.69	0.60	0.40	1
7	0.11	0.81	0.14	0.81	0.46	0.63	0.79	0.63	1.00	0.81	0.64	0.36	1
8	0.11	0.81	0.21	0.81	0.54	0.50	0.75	0.44	0.96	0.44	0.62	0.38	1
9	0.11	0.81	0.18	0.81	0.50	0.69	0.79	0.69	1.00	0.69	0.68	0.32	1
10	0.11	0.75	0.18	0.81	0.46	0.69	0.79	0.69	0.96	0.69	0.60	0.40	1
11	0.11	0.75	0.18	0.81	0.36	0.69	0.75	0.56	1.00	0.75	0.65	0.35	1
12	0.11	0.63	0.18	0.56	0.43	0.56	0.75	0.63	0.96	0.94	0.32	0.68	2
13	0.07	0.81	0.32	0.63	0.46	0.63	0.39	0.63	0.93	0.81	0.58	0.42	1
14	0.07	0.56	0.14	0.56	0.29	0.56	0.75	0.56	0.96	0.94	0.38	0.62	2
15	0.11	0.56	0.18	0.63	0.21	0.63	0.82	0.81	1.00	0.88	0.32	0.68	2
16	0.11	0.50	0.14	0.56	0.29	0.56	0.79	0.75	0.96	0.94	0.25	0.75	2
17	0.11	0.50	0.18	0.56	0.46	0.50	0.79	0.63	1.00	1.00	0.40	0.60	2
18	0.11	0.75	0.29	0.69	0.57	0.56	0.75	0.63	0.96	0.81	0.60	0.40	1
19	0.07	0.81	0.25	0.75	0.46	0.63	0.79	0.63	0.96	0.81	0.31	0.69	2
20	0.11	0.81	0.29	0.69	0.68	0.50	0.75	0.56	0.96	0.81	0.63	0.37	1
21	0.11	0.75	0.36	0.63	0.61	0.56	0.75	0.56	0.96	0.75	0.31	0.69	2
22	0.11	0.88	0.18	0.88	0.46	0.75	0.21	0.88	0.96	0.56	0.57	0.43	1
23	0.07	0.94	0.18	0.94	0.25	0.94	0.82	0.63	0.96	0.50	0.64	0.36	1
24	0.11	0.94	0.29	0.81	0.50	0.56	0.36	0.69	1.00	0.44	0.58	0.42	1
25	0.11	0.88	0.18	0.94	0.57	0.63	0.79	0.56	0.93	0.63	0.67	0.33	1
26	0.07	0.75	0.14	0.75	0.39	0.75	0.71	0.69	0.96	0.88	0.66	0.34	1
27	0.07	0.69	0.32	0.56	0.54	0.56	0.75	0.56	0.96	0.94	0.67	0.33	1
28	0.07	0.63	0.21	0.56	0.39	0.56	0.75	0.63	1.00	0.94	0.37	0.63	2
29	0.11	0.63	0.39	0.44	0.54	0.56	0.75	0.56	1.00	0.94	0.58	0.42	1
30	0.11	0.69	0.21	0.69	0.50	0.56	0.71	0.56	0.96	0.81	0.59	0.41	1
31	0.11	0.63	0.39	0.44	0.54	0.56	0.75	0.56	1.00	0.94	0.58	0.42	1
32	0.11	0.63	0.39	0.38	0.46	0.44	0.71	0.50	0.96	0.94	0.33	0.67	2
33	0.07	0.63	0.25	0.63	0.43	0.56	0.71	0.50	0.96	0.88	0.62	0.38	1
34	0.14	0.75	0.18	0.81	0.46	0.69	0.71	0.69	0.96	0.81	0.66	0.34	1
35	0.07	0.69	0.18	0.75	0.46	0.75	0.75	0.75	0.96	0.88	0.34	0.66	2
36	0.11	0.81	0.14	0.88	0.36	0.88	0.82	0.75	1.00	0.81	0.60	0.40	1
37	0.11	0.69	0.18	0.69	0.43	0.75	0.79	0.81	1.00	0.88	0.35	0.65	2
38	0.07	0.75	0.14	0.81	0.36	0.75	0.75	0.75	0.96	0.94	0.71	0.29	1
39	0.07	0.81	0.18	0.81	0.43	0.75	0.79	0.75	1.00	0.88	0.60	0.40	1
40	0.07	0.81	0.25	0.69	0.39	0.63	0.75	0.63	0.93	0.69	0.63	0.37	1
41	0.07	0.81	0.29	0.63	0.43	0.56	0.71	0.44	0.82	0.56	0.60	0.40	1
42	0.11	0.81	0.21	0.69	0.50	0.56	0.82	0.56	0.86	0.56	0.62	0.38	1
43	0.11	0.88	0.29	0.75	0.50	0.63	0.75	0.56	0.86	0.56	0.59	0.41	1
44	0.04	0.81	0.21	0.75	0.54	0.44	0.75	0.50	0.86	0.63	0.37	0.63	2
45	0.07	0.56	0.18	0.56	0.54	0.63	0.79	0.75	0.96	0.81	0.41	0.59	2
46	0.11	0.56	0.50	0.38	0.68	0.44	0.75	0.63	0.96	0.75	0.39	0.61	2
47	0.07	0.63	0.29	0.44	0.50	0.50	0.75	0.69	0.96	0.81	0.32	0.68	2
48	0.07	0.56	0.29	0.44	0.64	0.50	0.75	0.63	0.96	0.81	0.30	0.70	2
49	0.11	0.69	0.21	0.69	0.50	0.50	0.71	0.56	0.96	0.81	0.66	0.34	1
50	0.11	0.63	0.18	0.63	0.54	0.44	0.75	0.63	1.00	0.88	0.30	0.70	2

**Table 3:** Results of model identification of the left plantar pressure data.

Number	Training sample	Training number	Test sample	Test number	Identification accuracy
1	1–40	40	41–50	10	60%
2	1–30, 41–50	40	31–40	10	60%
3	1–20, 31–50	40	21–30	10	90%
4	1–10, 21–50	40	11–20	10	70%
5	11–50	40	1–10	10	100%

**Table 4:** Results of cluster analysis of plantar pressure data model from right side.

Number	*C*_1*x*_	*C*_1*y*_	*C*_2*x*_	*C*_2*y*_	*C*_3*x*_	*C*_3*y*_	*C*_4*x*_	*C*_4*y*_	*C*_5*x*_	*C*_5*y*_	Mode_1	Mode_2	Category
1	0.11	0.57	0.18	0.50	0.50	0.57	0.25	0.50	0.96	0.43	0.29	0.71	2
2	0.11	0.71	0.14	0.57	0.36	0.57	0.79	0.36	0.96	0.36	0.70	0.30	1
3	0.07	0.57	0.14	0.57	0.43	0.57	0.75	0.64	0.96	0.50	0.31	0.69	2
4	0.07	0.36	0.14	0.29	0.39	0.50	0.79	0.64	1.00	0.64	0.25	0.75	2
5	0.07	0.29	0.14	0.29	0.46	0.57	0.79	0.71	0.96	0.64	0.33	0.67	2
6	0.11	0.36	0.18	0.29	0.18	0.29	0.79	0.71	1.00	0.71	0.32	0.68	2
7	0.11	0.29	0.14	0.29	0.57	0.64	0.82	0.64	1.00	0.64	0.30	0.70	2
8	0.11	0.36	0.14	0.36	0.46	0.57	0.79	0.64	1.00	0.57	0.30	0.70	2
9	0.07	0.29	0.14	0.29	0.46	0.57	0.79	0.71	0.96	0.64	0.33	0.67	2
10	0.11	0.36	0.18	0.29	0.18	0.29	0.79	0.71	1.00	0.71	0.32	0.68	2
11	0.11	0.36	0.14	0.29	0.43	0.50	0.79	0.64	1.00	0.71	0.30	0.70	2
12	0.14	0.50	0.18	0.50	0.43	0.57	0.75	0.57	0.82	0.64	0.27	0.73	2
13	0.07	0.50	0.21	0.43	0.57	0.50	0.79	0.43	0.93	0.21	0.78	0.22	1
14	0.11	0.64	0.14	0.64	0.50	0.57	0.75	0.43	0.96	0.29	0.81	0.19	1
15	0.07	0.36	0.18	0.36	0.46	0.43	0.75	0.50	0.96	0.29	0.33	0.67	2
16	0.11	0.43	0.14	0.43	0.43	0.50	0.75	0.57	0.96	0.36	0.79	0.21	1
17	0.11	0.50	0.29	0.57	0.57	0.57	0.75	0.64	0.96	0.43	0.30	0.70	2
18	0.07	0.57	0.25	0.71	0.43	0.71	0.79	0.71	1.00	0.50	0.72	0.28	1
19	0.07	0.50	0.14	0.57	0.54	0.64	0.32	0.64	0.96	0.43	0.53	0.47	1
20	0.11	0.50	0.32	0.57	0.54	0.57	0.79	0.50	1.00	0.29	0.84	0.16	1
21	0.11	0.64	0.32	0.86	0.57	0.86	0.79	0.64	0.96	0.36	0.80	0.20	1
22	0.11	0.29	0.18	0.29	0.50	0.43	0.18	0.29	0.96	0.64	0.35	0.65	2
23	0.11	0.50	0.18	0.50	0.46	0.71	0.82	0.93	0.96	1.00	0.33	0.67	2
24	0.07	0.36	0.18	0.36	0.36	0.43	0.21	0.36	0.96	0.57	0.32	0.68	2
25	0.11	0.43	0.14	0.43	0.25	0.43	0.14	0.43	0.96	0.57	0.34	0.66	2
26	0.07	0.29	0.29	0.57	0.61	0.71	0.75	0.71	0.96	0.57	0.37	0.63	2
27	0.11	0.50	0.14	0.50	0.54	0.64	0.71	0.64	0.96	0.50	0.49	0.51	2
28	0.07	0.43	0.18	0.43	0.61	0.71	0.75	0.64	0.96	0.57	0.32	0.68	2
29	0.11	0.43	0.25	0.57	0.64	0.64	0.79	0.64	0.96	0.57	0.37	0.63	2
30	0.07	0.43	0.25	0.57	0.61	0.71	0.75	0.71	0.96	0.57	0.74	0.26	1
31	0.07	0.43	0.25	0.50	0.46	0.50	0.82	0.57	0.96	0.57	0.38	0.62	2
32	0.11	0.29	0.18	0.29	0.50	0.50	0.79	0.64	0.96	0.79	0.29	0.71	2
33	0.07	0.29	0.25	0.43	0.43	0.43	0.82	0.57	0.96	0.64	0.70	0.30	1
34	0.11	0.36	0.29	0.50	0.50	0.57	0.82	0.57	0.96	0.64	0.89	0.11	1
35	0.07	0.43	0.18	0.43	0.54	0.50	0.79	0.57	0.96	0.57	0.87	0.13	1
36	0.07	0.29	0.18	0.36	0.43	0.43	0.75	0.64	0.96	0.57	0.80	0.20	1
37	0.11	0.36	0.18	0.43	0.43	0.57	0.79	0.71	0.96	0.71	0.26	0.74	2
38	0.07	0.50	0.21	0.50	0.57	0.64	0.50	0.71	0.96	0.43	0.49	0.51	2
39	0.07	0.57	0.39	0.79	0.57	0.71	0.75	0.64	0.96	0.43	0.61	0.39	1
40	0.11	0.50	0.18	0.57	0.57	0.64	0.75	0.64	0.96	0.36	0.70	0.30	1
41	0.07	0.43	0.21	0.50	0.46	0.64	0.75	0.64	0.96	0.29	0.76	0.24	1
42	0.07	0.43	0.25	0.57	0.57	0.64	0.75	0.57	0.96	0.29	0.80	0.20	1
43	0.11	0.50	0.21	0.57	0.61	0.71	0.79	0.64	1.00	0.43	0.65	0.35	1
44	0.07	0.43	0.14	0.43	0.46	0.57	0.79	0.64	1.00	0.57	0.33	0.67	2
45	0.07	0.50	0.14	0.50	0.36	0.50	0.75	0.64	0.96	0.50	0.27	0.73	2
46	0.11	0.64	0.21	0.64	0.43	0.71	0.75	0.71	0.96	0.43	0.37	0.63	2
47	0.11	0.50	0.21	0.50	0.54	0.64	0.79	0.57	0.96	0.50	0.91	0.09	1
48	0.11	0.43	0.21	0.43	0.61	0.57	0.82	0.50	1.00	0.43	0.74	0.26	1
49	0.11	0.43	0.18	0.43	0.57	0.64	0.75	0.71	0.96	0.36	0.31	0.69	2
50	0.11	0.50	0.29	0.57	0.54	0.71	0.79	0.79	1.00	0.57	0.81	0.19	1

**Table 5:** Results of model identification of the right plantar pressure data.

Number	Training sample	Training number	Test sample	Test number	Identification accuracy
1	1–40	40	41–50	10	70%
2	1–30, 41–50	40	31–40	10	40%
3	1–20, 31–50	40	21–30	10	60%
4	1–10, 21–50	40	11–20	10	50%
5	11–50	40	1–10	10	90%

## References

[1] Eisenhardt J. R., Cook D., Pregler I., Foehl H. C. (1996). Changes in temporal gait characteristics and pressure distribution for bare feet versus various heel heights. *Gait and Posture*.

[2] Minns R. J., Craxford A. D. (1984). Pressure under the forefoot in rheumatoid arthritis. A comparison of static and dynamic methods of assessment. *Clinical Orthopaedics and Related Research*.

[3] Abboud R. J., Rowley D. I., Newton R. W. (2000). Lower limb muscle dysfunction may contribute to foot ulceration in diabetic patients. *Clinical Biomechanics*.

[4] Stokes I. A. F., Hutton W. C., Evans M. J. (1975). The effects of hallux valgus and Keller's operation on the load-bearing function of the foot during walking. *Acta Orthopaedica Belgica*.

[5] Stolwijk N. M., Duysens J., Louwerens J. W. K., Keijsers N. L. W. (2010). Plantar pressure changes after long-distance walking. *Medicine and Science in Sports and Exercise*.

[6] Stolwijk N. M., Duysens J., Louwerens J. W. K., van de Ven Y. H. M., Keijsers N. L. W. (2013). Flat feet, happy feet? Comparison of the dynamic plantar pressure distribution and static medial foot geometry between Malawian and Dutch adults. *PLoS ONE*.

[7] Pataky T. C., Maiwald C. (2011). Spatiotemporal volumetric analysis of dynamic plantar pressure data. *Medicine and Science in Sports and Exercise*.

[8] Taylor A. J., Menz H. B., Keenan A.-M. (2004). The influence of walking speed on plantar pressure measurements using the two-step gait initiation protocol. *The Foot*.

[9] Lai Y.-C., Lin H.-S., Pan H.-F., Chang W.-N., Hsu C.-J., Renn J.-H. (2014). Impact of foot progression angle on the distribution of plantar pressure in normal children. *Clinical Biomechanics*.

[10] Ruspini E. H. (1969). A new approach to clustering. *Information and Control*.

[11] Backer E., Jain A. K. (1981). Clustering performance measure based on fuzzy set decomposition. *IEEE Transactions on Pattern Analysis and Machine Intelligence*.

[12] Horta D., de Andrade I. C., Campello R. J. G. B. (2011). Evolutionary fuzzy clustering of relational data. *Theoretical Computer Science*.

[13] Li X.-L., Liu D.-X., Jia C., Chen X.-Z. (2015). Multi-model control of blast furnace burden surface based on fuzzy SVM. *Neurocomputing*.

[14] Bezdek J. C. (1981). *Pattern Recognition with Fuzzy Objective Function Algorithm*.

[15] Cortes C., Vapnik V. (1995). Support-vector networks. *Machine Learning*.

[16] Wang W., Li X. L. (2001). *Multiple Model Adaptive Control*.

[17] Li X. L., Zhang W. (2010). Multiple model iterative learning control. *Neurocomputing*.

[18] Keijsers N. L. W., Stolwijk N. M., Nienhuis B., Duysens J. (2009). A new method to normalize plantar pressure measurements for foot size and foot progression angle. *Journal of Biomechanics*.

[19] Hartigan J. A., Wong M. A. (1979). Algorithm AS 136: a K-means clustering algorithm. *Applied Statistics*.

[20] Andriacchi T. P., Dyrby C. O. (2005). Interactions between kinematics and loading during walking for the normal and ACL deficient knee. *Journal of Biomechanics*.

[21] Defrate L. E., Papannagari R., Gill T. J., Moses J. M., Pathare N. P., Li G. (2006). The 6 degrees of freedom kinematics of the knee after anterior cruciate ligament deficiency: an in vivo imaging analysis. *The American Journal of Sports Medicine*.

[22] Ikeda H., Kurosawa H., Kim S.-G. (2002). Quadriceps torque curve pattern in patients with anterior cruciate ligament injury. *International Orthopaedics*.

[23] Chiu M.-C., Wu H.-C., Chang L.-Y., Wu M.-H. (2013). Center of pressure progression characteristics under the plantar region for elderly adults. *Gait and Posture*.

[24] Hongshi H., Yuanyuan Y., Qinwei G., Yan X., Yingfang A. (2015). Temporal characteristics of plantar pressure variables during walking following anterior cruciate ligament rupture. *Chinese Journal of Sports Medicine*.

